# Detection of SARS-CoV-2 Among Residents and Staff Members of an Independent and Assisted Living Community for Older Adults — Seattle, Washington, 2020

**DOI:** 10.15585/mmwr.mm6914e2

**Published:** 2020-04-10

**Authors:** Alison C. Roxby, Alexander L. Greninger, Kelly M. Hatfield, John B. Lynch, Timothy H. Dellit, Allison James, Joanne Taylor, Libby C. Page, Anne Kimball, Melissa Arons, Laura A. Schieve, Albert Munanga, Nimalie Stone, John A. Jernigan, Sujan C. Reddy, James Lewis, Seth A. Cohen, Keith R. Jerome, Jeffrey S. Duchin, Santiago Neme

**Affiliations:** ^1^Department of Medicine, University of Washington, Seattle; ^2^Department of Global Health, University of Washington, Seattle; ^3^Department of Laboratory Medicine, University of Washington, Seattle; ^4^CDC COVID-19 Response Team; ^5^Public Health – Seattle & King County, Washington; ^6^Department of Biobehavioral Nursing and Health Informatics, University of Washington School of Nursing, Seattle; ^7^Era Living Retirement Communities, Seattle, Washington;^ 8^Vaccine and Infectious Disease Division, Fred Hutchinson Cancer Research Center, Seattle, Washington.

In the Seattle, Washington metropolitan area, where the first case of novel coronavirus 2019 disease (COVID-19) in the United States was reported (*1*), a community-level outbreak is ongoing with evidence of rapid spread and high morbidity and mortality among older adults in long-term care skilled nursing facilities (SNFs) (*2*,*3*). However, COVID-19 morbidity among residents of senior independent and assisted living communities, in which residents do not live as closely together as do residents in SNFs and do not require skilled nursing services, has not been described. During March 5–9, 2020, two residents of a senior independent and assisted living community in Seattle (facility 1) were hospitalized with confirmed COVID-19 infection; on March 6, social distancing and other preventive measures were implemented in the community. UW Medicine (the health system linked to the University of Washington), Public Health – Seattle & King County, and CDC conducted an investigation at the facility. On March 10, all residents and staff members at facility 1 were tested for SARS-CoV-2, the virus that causes COVID-19, and asked to complete a questionnaire about their symptoms; all residents were tested again 7 days later. Among 142 residents and staff members tested during the initial phase, three of 80 residents (3.8%) and two of 62 staff members (3.2%) had positive test results. The three residents had no symptoms at the time of testing, although one reported an earlier cough that had resolved. A fourth resident, who had negative test results in the initial phase, had positive test results 7 days later. This resident was asymptomatic on both days. Possible explanations for so few cases of COVID-19 in this residential community compared with those in several Seattle SNFs with high morbidity and mortality include more social distancing among residents and less contact with health care providers. In addition, early implementation of stringent isolation and protective measures after identification of two COVID-19 cases might have been effective in minimizing spread of the virus in this type of setting. When investigating a potential outbreak of COVID-19 in senior independent and assisted living communities, symptom screening is unlikely to be sufficient to identify all persons infected with SARS-CoV-2. Adherence to CDC guidance to prevent COVID-19 transmission in senior independent and assisted living communities (*4*) could be instrumental in preventing a facility outbreak.

Facility 1 comprises 83 apartments (45 independent living and 38 assisted living) along multiple hallways; and communal dining, library, and activity areas. Residents are physically able to move about the facility with minimal assistance. Independent-living residents have access to help if needed but are otherwise unaided; assisted-living residents have daily in-home help with medications and activities of daily living.

All residents were able to leave their rooms and move about the facility until March 6, when social distancing and other preventive measures were implemented. Residents were isolated in their rooms with no communal meals or activities, no visitors were allowed in the facility, and staff member screening and exclusion of symptomatic staff members were implemented. Enhanced hygiene practices were put into effect, including cleaning and disinfection of frequently touched surfaces and additional hand hygiene stations in hallways for workers to use.

All residents and staff members participated in this investigation with the exception of the two hospitalized residents with COVID-19 and one resident staying with relatives off-site for an extended period. Two rounds of SARS-CoV-2 testing were conducted, 7 days apart. On the day of the first round of testing, March 10, social distancing and other preventive measures had been in effect for >72 hours. Nasopharyngeal swabs were used to collect specimens from all residents and staff members; SARS-CoV-2 real-time reverse transcription–polymerase chain reaction assay was performed on specimens. Residents and staff members were also asked to complete a questionnaire assessing fever, cough, and other symptoms during the preceding 14 days; some residents received assistance from staff members to complete the questionnaire. Staff members from all shifts came to the facility for the assessment, including two ill staff members who were tested in their cars. In addition, specimens and symptom questionnaires were collected on March 11 from two residents who had been off-site and from several staff members who had been unable to go to the facility on March 10. All residents were tested again 7 days later; symptom information was not collected at that time, with the exception of symptom ascertainment through follow-up of any resident with a positive test result. Staff members were not retested because they had no new facility exposure to SARS-CoV-2; all residents who had positive test results during the first round were in isolation, and the facility’s personal protective equipment protocols* were being followed. Testing procedures for the second round were the same as those used for the first round.

In total, 80 residents and 62 staff members were tested on March 10 and 11. Mean age of residents was 86 years (range = 69–102 years); 77% were female; and 79% had one or more chronic medical conditions including chronic lung disease, diabetes mellitus, cardiovascular disease, cerebrovascular disease, renal disease, cognitive impairment, or obesity. Mean age of staff members was 40 years (range = 16–70 years), and 72% were female.

SARS-CoV-2 was detected in three (3.8%) residents and two (3.2%) staff members (Table). None of the residents with positive tests reported symptoms at the time of testing; however, one (resident C) reported resolved mild cough and loose stool during the preceding 14 days. All three residents with positive test results were living on separate floors in their own apartments; one received assistance with activities of daily living. One resident lived on the same floor as the two hospitalized residents with known COVID-19, and one had known close contact with one of the hospitalized residents; the third resident who had positive test results had no contact with either of the hospitalized residents. One staff member who had positive test results for SARS-CoV-2 worked in dining services, and the other worked as a health aide. Both reported symptoms. One staff member (staff member D) reported headache for 10 days, and the other (staff member E) reported a 5-day history of body aches, headache, and cough; this staff member had not worked while ill. When the second round of testing was conducted 7 days later, one additional positive test result was reported for an asymptomatic resident who had negative test results on the first round.

**TABLE:** Characteristics of residents and staff members with positive SARS-CoV-2 test results* on day 1 and day 7 — independent and assisted living community for older adults, Seattle, Washington, March 10 and 17, 2020

Test group/Case ID	Sex	Age (yrs)	Symptoms reported in 14 days preceding first test	SARS-CoV-2 test results	
				Day 1	Day 7
**Persons with positive test results on day 1**					
Resident A	Female	92	None	Positive	Negative
Resident B	Female	82	None	Positive	Positive
Resident C	Male	75	Cough (resolved) and one loose stool on day of test	Positive	Positive
Staff member D	Female	24	Headache x 10 days	Positive	Not retested
Staff member E	Female	51	Body aches, cough, and headache x 5 days	Positive	Not retested
**Person with positive test result on day 7**					
Resident F	Female	86	None	Negative	Positive

* Defined as a real-time reverse transcription–polymerase chain reaction testing cycle threshold value <40.

During the first round of testing and symptom screening, symptoms were reported by 42% of residents and 25% of staff members who had negative test results for SARS-CoV-2. Symptoms reported by residents who had negative test results included sore throat, chills, confusion, body aches, dizziness, malaise, headaches, cough, shortness of breath, and diarrhea. Signs and symptoms reported by staff members who had negative test results included fever, sore throat, chills, confusion, malaise, headache, cough, and diarrhea. All residents remained in the independent and assisted living facility in isolation and were clinically stable (i.e., no change in their usual state of health) as of March 31.

## Discussion

In this senior independent and assisted living facility, symptom screening of residents did not identify persons who had positive test results for SARS-CoV-2; three of the four residents who had positive test results were asymptomatic at the time of testing, and one reported a cough that had resolved. Moreover, >40% of residents who had test results (whether positive or negative) reported one or more symptoms potentially compatible with COVID-19 during the preceding 2 weeks.

That only four residents had positive test results differed markedly from reports from two Seattle SNFs that experienced high COVID-19 transmission, morbidity, and mortality (*2*,*3*). Possible explanations for differences in findings in this residential community from those in SNFs include more social distancing among residents and less contact with health care providers in independent and assisted living communities than that in SNFs. In addition, early implementation of stringent isolation and protective measures after identification of two COVID-19 cases might have been effective in minimizing spread of the virus.

The findings in this report are subject to at least one limitation. Symptom reports by residents and staff members might have been subject to recall bias, given the general anxiety about COVID-19 in response to the identification of the two initial COVID-19 cases. Nonetheless, the high percentage of both residents and staff members who had negative test results for SARS-CoV-2, yet reported symptoms, illustrates the limitations associated with COVID-19 case identification strategies determined by presence of symptoms alone. The findings from this investigation underscore the importance of SARS-CoV-2 mitigation measures, including social distancing, visitor restriction, resident and staff member testing, exclusion of ill staff members, and enhanced disinfection and hygiene practices, which are consistent with current CDC guidance for preventing transmission of COVID-19 in independent and assisted living communities (*4*).

**Summary**

**What is already known about this topic?**

Community transmission of COVID-19 has been associated with rapid spread and high morbidity and mortality among older adults in long-term skilled nursing facilities. COVID-19 transmission in other types of senior living communities has not been described.

**What is added by this report?**

Following identification of two COVID-19 cases in a Seattle independent and assisted living facility, stringent preventive measures were implemented. Testing of all residents and staff members found few cases of COVID-19. Three of four residents who had positive test results were asymptomatic.

**What are the implications for public health practice?**

Symptom-based screening might not identify SARS-CoV-2 infections in independent and assisted living facility residents, underscoring the importance of adhering to CDC guidance to prevent COVID-19 transmission in senior living communities.

## Footnotes

* Current CDC recommendations on use of personal protective equipment by health care personnel caring for patients with suspected or confirmed COVID-19 are available at https://www.cdc.gov/coronavirus/2019-ncov/infection-control/control-recommendations.html.
